# Epoetin beta pegol alleviates oxidative stress and exacerbation of renal damage from iron deposition, thereby delaying CKD progression in progressive glomerulonephritis rats

**DOI:** 10.14814/phy2.12637

**Published:** 2015-12-03

**Authors:** Michinori Hirata, Yoshihito Tashiro, Ken Aizawa, Ryohei Kawasaki, Yasushi Shimonaka, Koichi Endo

**Affiliations:** ^1^ Product Research DepartmentChugai Pharmaceutical Co., Ltd.GotembaShizuokaJapan

**Keywords:** CERA, epoetin beta pegol (continuous erythropoietin receptor activator, hepcidin‐25, iron deposition, liver‐type fatty acid–binding protein (L‐FABP)

## Abstract

The increased deposition of iron in the kidneys that occurs with glomerulopathy hinders the functional and structural recovery of the tubules and promotes progression of chronic kidney disease (CKD). Here, we evaluated whether epoetin beta pegol (continuous erythropoietin receptor activator: CERA), which has a long half‐life in blood and strongly suppresses hepcidin‐25, exerts renoprotection in a rat model of chronic progressive glomerulonephritis (cGN). cGN rats showed elevated urinary total protein excretion (uTP) and plasma urea nitrogen (UN) from day 14 after the induction of kidney disease (day 0) and finally declined into end‐stage kidney disease (ESKD), showing reduced creatinine clearance with glomerulosclerosis, tubular dilation, and tubulointerstitial fibrosis. A single dose of CERA given on day 1, but not on day 16, alleviated increasing uTP and UN, thereby delaying ESKD. In the initial disease phase, CERA significantly suppressed urinary 8‐OHdG and liver‐type fatty acid–binding protein (L‐FABP), a tubular damage marker. CERA also inhibited elevated plasma hepcidin‐25 levels and alleviated subsequent iron accumulation in kidneys in association with elevated urinary iron excretion and resulted in alleviation of growth of Ki67‐positive tubular and glomerular cells. In addition, at day 28 when the exacerbation of uTP occurs, a significant correlation was observed between iron deposition in the kidney and urinary L‐FABP. In our study, CERA mitigated increasing kidney damage, thereby delaying CKD progression in this glomerulonephritis rat model. Alleviation by CERA of the exacerbation of kidney damage could be attributable to mitigation of tubular damage that might occur with lowered iron deposition in tubules.

## Introduction

Mesangioproliferative glomerulonephritis is a common type of glomerulonephritis and a major contributor to end‐stage kidney disease worldwide (Barratt and Feehally 2006). In IgA nephropathy—a representative form of mesangioproliferative glomerulonephritis—deposition of iron is observed in the kidneys in more 30% of patients (Wang et al. 2001), causing incomplete recovery of renal function (Gutierrez et al. 2012; Moreno et al. 2012). In rat models of nephropathy, too, iron accumulation is also observed in the tubular cells (Izuhara et al. 2005; Naito et al. 2012), which influences both the functional and structural damage of the tubules (Harris et al. 1994; Nankivell et al. 1994). Thus, the increased deposition of iron in the kidneys that occurs with glomerulopathy may hinder the functional and structural recovery of the tubules and promotes progression of chronic kidney disease (CKD).

The renoprotective effects of erythropoiesis stimulating agents (ESAs) in animal models possibly involve antiapoptotic effects (Sharples et al. 2004) and acceleration of tubular regeneration (Johnson et al. 2006). Darbepoetin‐*α*, a long‐acting erythropoietin (EPO), acts to reduce glomerulosclerosis by accelerating glomerular regeneration (Canadillas et al. 2010). Epoetin beta pegol (continuous erythropoietin receptor activator: CERA; methoxy polyethylene glycol‐epoetin beta), which has a longer half‐life than other erythropoietin stimulating agents (ESAs) and stronger suppression of hepcidin‐25 than EPO (Sasaki et al. 2012), has also been shown to confer renoprotective effects in acute kidney disease models (Aizawa et al. 2012, 2014; Rodrigues et al. 2012). However, the renoprotective effect of CERA on progression of CKD still remains unclear.

The irreversible model of anti‐Thy‐1 nephritis shows progressive glomerulosclerosis and tubulointerstitial fibrosis and is regarded as the model that most closely mimics human mesangioproliferative glomerulonephritis, such as IgA nephropathy (Ostendorf et al. 2001). It has been demonstrated that the irreversibility of kidney disease in this model is determined in the acute phase (Ostendorf et al. 2001; Tsuji et al. 2006). Therefore, this model may be useful for evaluating factors exacerbating kidney damage and for exploring crucial factors leading to progression of CKD.

The aims of the present study were (1) to assess whether CERA administered to cGN rats in the acute phase could protect against development of CKD; and (2) to explore the underlying mechanisms by which CERA delayed progression of CKD from the standpoint of iron‐related tubular damage.

## Materials and Methods

### Animal models

Male Fisher 344 rats (95–115 g) were obtained from Charles River Japan (Atsugi, Japan). The animals were raised on standard rodent chow (CE‐2; Japan Clea, Tokyo, Japan) and given free access to tap water until 7 weeks of age. The animals were housed in a constant‐temperature room with a 12‐h dark/12‐h light cycle and 35–75% relative humidity. Chronic glomerulonephritis (cGN) rats were produced by intravenous injection of a mouse anti‐rat CD90 (Thy1.1) monoclonal antibody (Cedarlane Labs, Ontario, Canada) dissolved in PBS at a single dose of 0.6 mg/kg body weight immediately after left kidney nephrectomy (day 0). Sham‐operated (Sham) rats underwent surgical manipulation without any removal of the kidney. Sham rats were injected with PBS (vehicle) instead of the antibody. All animal procedures were conducted in accordance with Chugai Pharmaceutical's ethical guidance for animal care, and all experimental protocols were approved by the Animal Care Committee of the institution and conformed to the *Guide for the Care and Use of Laboratory Animals* published by the US National Institutes of Health.

### Experimental protocols

#### Experimental protocol 1: The effect of CERA on CKD progression in cGN rats

On day 0, the animals were randomly divided into three groups: a vehicle‐treated (PBS‐treated) cGN group (cGN + V; *n *=* *12), a group of a single dose of CERA given on day 1 (cGN + C; *n *=* *12), and a group of a single dose of CERA given on day 16 (*n *=* *10). On day 1 or 16, CERA (Roche, Basel, Switzerland) was administered at a dose of 25 *μ*g/kg body weight. Vehicle was administered intravenously in a vehicle‐treated (PBS‐treated) cGN group. For the evaluation of renoprotection over time, 24‐h urinary samples were collected by using metabolic cages at weeks 1, 2, 4, 6, 8, 12, 16, and 20. At the same time, heparinized blood samples were obtained from the jugular veins of anesthetized rats. All samples were stored at −30°C until measurement of parameters. At week 20, after the measurement of blood pressure, the animals were euthanized with collecting blood from the abdominal aorta and kidney weights were measured. Then, each remnant kidney was divided for histopathological analysis and for evaluation of iron content.

#### Experimental protocol 2: Exploration of mechanisms underlying the renoprotective effect of CERA in cGN rats

Preparation of cGN rats was the same as in *Experimental Protocol 1*. After a single dose of CERA or vehicle given on day 1, urine and plasma samples were collected on days 1, 4, 8, 14, and 28 (*n *=* *8–12). Rats were euthanized at each sampling point, and each remnant kidney was divided for histopathological and immunohistochemical analyses measuring iron content. To evaluate normal levels, Sham rats (*n *=* *5–6) were euthanized on day 1.

### Biological parameters

At each of the sampling points in *Experimental Protocol 1*, plasma urea nitrogen (UN), plasma creatinine (Cr), plasma iron, urinary total protein (uTP), urinary creatinine, and urinary iron were measured with an automatic analyzer (7170S; Hitachi High‐Technologies Corporation, Tokyo, Japan). In *Experimental Protocol 2*, plasma iron and urinary iron were measured with the automatic analyzer. Urinary liver‐type fatty acid–binding protein (L‐FABP) was measured by ELISA (R&D Systems, Minneapolis, MN). Urinary 8‐OHdG levels were determined using an ELISA kit (Japan Institute for the Control Aging, NIKKEN SEIL Co., Ltd, Shizuoka Japan). At week 20, creatinine clearance (CCr) was calculated on the basis of plasma and urinary Cr concentrations and the corresponding urine volume. Hemoglobin (Hb) levels were measured by hematology analyzer (XT‐2000iV; Sysmex Corporation, Kobe, Japan).

Plasma levels of hepcidin‐25 were quantified by liquid chromatography tandem mass spectrometry (LC‐MS/MS). Samples were introduced to a PLRP‐S column (300Å, 5 *μ*m, 150 × 2.1 mm; Agilent Technologies, Palo Alto, CA) and measured by LC‐MS/MS (API 5000; AB Sciex, Framingham, MA). Iron content in the kidney was measured by inductively coupled plasma optical emission spectroscopy (Optima 4300 DV; PerkinElmer, CT, Shelton). Systolic blood pressure was measured before euthanasia by tail‐cuff plethysmography (BP‐98A; Softron, Tokyo, Japan) in conscious trained rats. The means of five measurements for each animal were calculated.

### Histomorphometrical analysis

Kidney tissues were fixed in 10% neutral buffered formalin, embedded in paraffin, and cut into 4‐*μ*m‐thick sections. The sections were stained with the periodic acid–Schiff reaction to determine glomerulosclerosis index and tubular dilation and with Masson's trichrome staining for tubulointerstitial fibrosis. Glomerulosclerosis index was assessed in 50 randomly selected glomeruli using a semiquantitative method as a described previously (Raij et al. 1984). Tubular dilation and tubulointerstitial fibrosis scores were quantified and rated according to morphological changes by using the following grades: 0, none; 1, minor; 2, mild; 3, moderate; 4, severe. Ferric iron deposits were stained using Berlin blue staining. All histological studies were performed in a blinded fashion.

### Immunohistochemical analysis

Tissue sections were deparaffinized in xylene and rehydrated in graded alcohol. Immunostaining for Ki67 was carried out by using a DAKO LSAB2 kit (K0609) according to the manufacturer's instructions. Briefly, tissue sections were heated at 121°C for 15 min using an autoclave, and citrate buffer (pH 6.0) was used for antigen retrieval. The sections were treated for 30 min with methanol to which 1% H_2_O_2_ was added to inactivate endogenous peroxidase activity. The sections were then incubated for 30 min at room temperature with anti‐Ki67 (M7248; DAKO) diluted 1:50 by DAKO Antibody Diluent (S2022; DAKO). After washing, slides were incubated with biotinylated anti‐mouse immunoglobulins and horseradish‐conjugated streptavidin (LSAB2, K0609; DAKO), each for 10 min. Then, DAB substrate (K3466; DAKO) was applied to develop the stain. Sections were washed and counterstained with hematoxylin. Ki67‐positive cells were counted in 50 randomly selected glomeruli and in the tubular epithelium of the renal cortex in 20 microscope fields per section at ×400 magnification as described in a previous paper (Johnson et al. 2006).

### Statistical analysis

All values are expressed as a mean ± SEM. Intergroup comparisons were assessed by unpaired *t*‐test, or data obtained over time were assessed by unpaired *t*‐test followed by Bonferroni post hoc test. Nonparametric data of histopathological analysis were assessed by Wilcoxon followed by Bonferroni post hoc test. uTP and UN data were assessed by two‐way ANOVA followed by Bonferroni post hoc test. *P*‐values of <0.05 were considered statistically significant. All statistical calculations were performed using SAS version 8.2 software (SAS Institute, Cary, NC). Statistical analysis for correlation between L‐FABP and kidney iron deposition was performed using JMP version 11 software (SAS Institute, Cary, NC).

## Results

### Renoprotective effect of CERA in rats with progressive kidney disease

The induction of kidney disease by injection of anti‐Thy1.1 antibody starts with mesangiolysis, and the insult lasts for several days. Because CERA injection was given on day 1 after the injection of anti‐Thy1.1 antibody in our protocol, we evaluated whether CERA affected the insult itself. We found that there was no significant difference in mesangiolysis index between the cGN + V group and the cGN + C group from day 2 to day 8 (data not shown). Therefore, CERA does not appear to suppress the insult itself.

#### Biological analysis of kidney function

**Figure 1:**
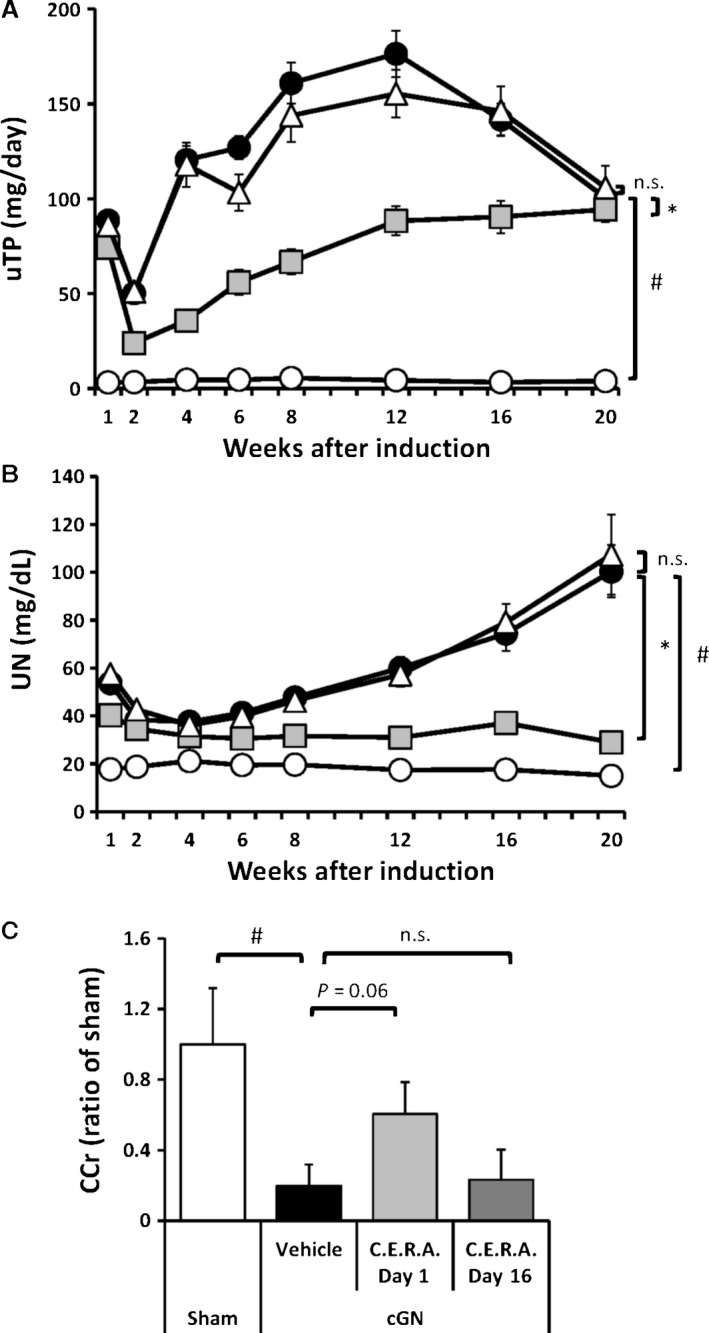
Changes in (A) urinary total protein excretion (uTP) and (B) plasma urea nitrogen (UN) over time until week 20. (C) Creatinine clearance (CCr) at week 20. Sham, sham‐operated normal control rats (white symbols and bars); vehicle, vehicle‐treated cGN rats (black symbols and bars); CERA day 1, CERA‐treated cGN rats on day 1 (light gray symbols and bars); CERA day 16, CERA‐treated cGN rats on day 16 (triangle symbols and dark bars). #*P* < 0.05 versus Sham control (*n* = 6); *P < 0.05 versus cGN rats (*n* = 6–12); n.s., not significance. Values are expressed as mean ± SEM.

uTP (Fig. 1A) in cGN rats instantly increased, with elevated levels measured at week 1; levels decreased again at week 2, but did not completely return to the normal range. In subsequent weeks, the levels of uTP deteriorated once more. Similarly, UN (Fig. 1B) in cGN rats was changed. In addition, the kidney function of cGN rats finally declined into end‐stage kidney disease as shown in the deterioration of CCr (Fig. 1C). A single dose of CERA given on day 1 significantly alleviated the exacerbation of uTP and UN seen after week 2 and resulted in suppressing the decline of CCr in cGN rats. However, a single dose of CERA given on day 16 did not suppress the exacerbation of uTP and UN and the decline of CCr (Fig. 1). Wet kidney weight as a proportion of body weight was significantly higher in the cGN + V group than in the Sham group, whereas this elevation was significantly suppressed in the cGN + C group at the end of the study (Table 1). In addition, Hb levels were significantly lower in the cGN + V group than in the Sham group. CERA treatment significantly prevented this decline as a result of preserving kidney function, although Hb levels showed a temporary elevation on day 15 (cGN + V: 12.0 ± 0.28 g/dL; cGN + C: 16.7 ± 0.19 g/dL, *P* < 0.05). There was significant difference in systolic blood pressure or in body weight between the cGN + V group and the cGN + C group (Table 1).

**Table 1:** Effect of CERA on body weight, kidney weight, hemoglobin, and systolic blood pressure at week 20

	Sham	cGN + Vehicle	cGN + CERA
BW, g	344.6 ± 7.1	294.7 ± 8.5†	329.1 ± 1.4*
KW/BW, mg/g	2.9 ± 0.05	6.7 ± 0.16†	5.4 ± 0.11*
Hb, g/dL	15.4 ± 0.3	12.1 ± 0.4†	14.3 ± 0.2*
SBP, mmHg	125.9 ± 1.2	154.4 ± 3.8†	140.1 ± 2.7*

BW, body weight; KW, kidney weight; SBP, systolic blood pressure; Hb, hemoglobin. Values are mean ± SEM, *n* = 6–12, ^†^ *P *<* *0.05 versus Sham, *P <* *0.05 versus cGN + Vehicle.

#### Renal morphology

**Figure 2:**
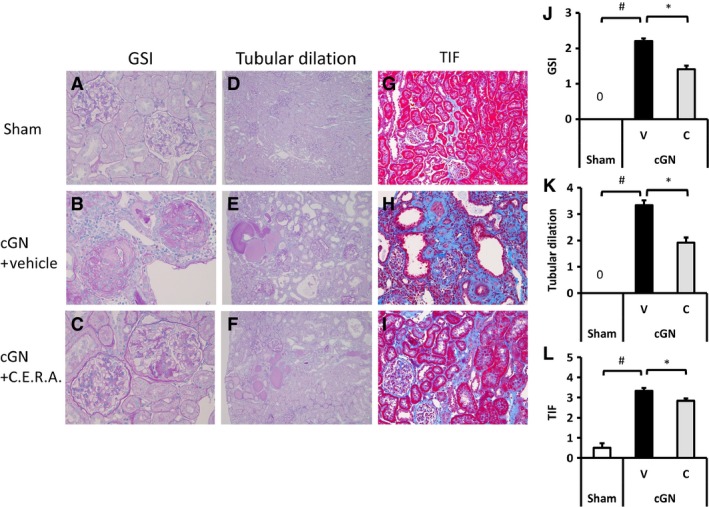
Histopathological analysis of changes in morphology of kidneys at week 20. Representative histological findings with periodic acid–Schiff (PAS) staining (A–F) for determining glomerulosclerosis index (GSI) and tubular dilation and Masson's trichrome staining (G–I) for determining tubulointerstitial fibrosis (TIF). Images are of Sham rats (A, D, G), cGN + vehicle (B, E, H), and cGN + CERA (C, F, I). Sham, sham‐operated control rats (white bars); V, vehicle‐treated cGN rats (black bars); C, CERA‐treated cGN rats (gray bars). Magnification:×80 (A–C);×280 (D–I). #*P* < 0.05 versus Sham control; *P < 0.05 versus cGN rats (*n* = 8–10). Values are expressed as mean ± SEM.

Renal histology as assessed by periodic acid–Schiff staining revealed that cGN rats had severe glomerulosclerosis and tubular dilation (Fig. 2); these changes were significantly attenuated in the cGN + C group as compared with in the cGN + V group. In addition, tubulointerstitial fibrosis (TIF) as assessed by Masson's trichrome staining was increased in cGN rats as compared with Sham rats and was significantly prevented by CERA treatment.

### Effect of CERA in the acute phase in rats with progressive kidney disease

Because the results of Experimental Protocol 1 showed that CERA treatment of the initial renal insult might be effective in delaying CKD progression, we next evaluated the effect of CERA on the initial changes in renal morphology and on iron‐related parameters until day 28.

#### Histopathological changes

**Figure 3:**
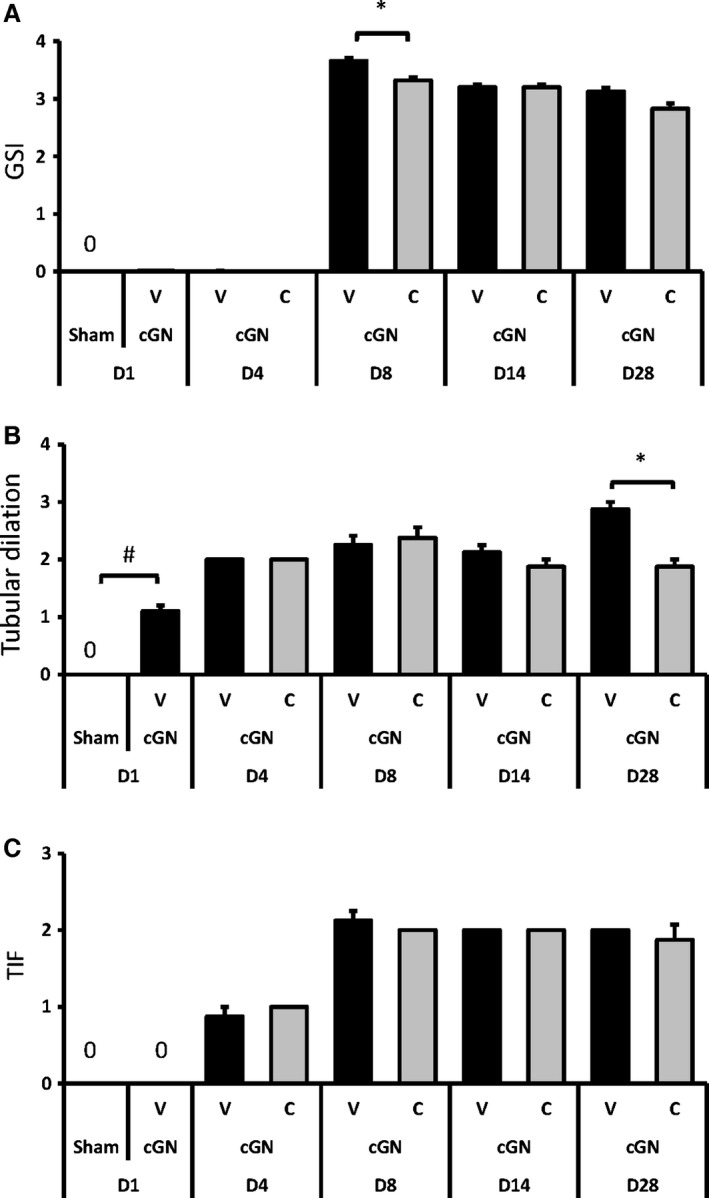
Histopathological analysis of initial changes in morphology of kidneys from day 1 (D1) to day 28 (D28). Sham, sham‐operated control rats (white bars); V, vehicle‐treated cGN rats (black bars); C, CERA‐treated cGN rats (gray bars); GSI, glomerulosclerosis index; TIF, tubulointerstitial fibrosis. #*P* < 0.05 versus Sham control; *P < 0.05 versus cGN rats (*n* = 8–10). Values are expressed as mean ± SEM.

Glomerulosclerosis index was clearly increased from day 8 to day 28, and CERA treatment significantly suppressed the elevation on day 8 (Fig. 3A). On the other hand, tubular dilation score was elevated at day 1, remained high until day 14, and then was observed to have worsened at day 28. CERA treatment significantly suppressed the deterioration observed on day 28 (Fig. 3B). TIF score was clearly increased from day 4 to day 28, but CERA did not affect the elevation during that period (Fig. 3C).

#### Changes in urinary oxidative stress markers

**Figure 4:**
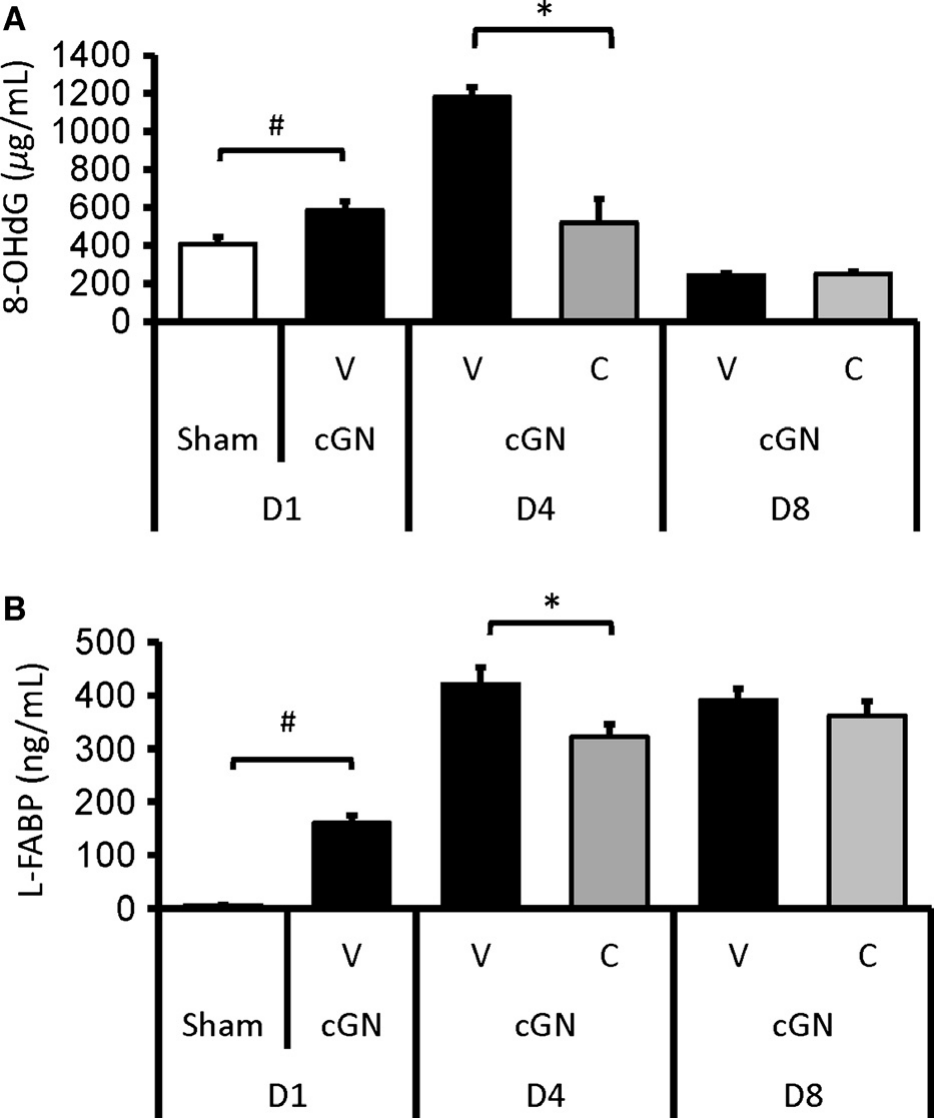
The effect of CERA on changes in (A) urinary 8‐OHdG levels and (B) urinary L‐FABP levels, from day 1 (D1) to day 8 (D8). Sham, sham‐operated control rats (white bars); V, vehicle‐treated cGN rats (black bars); C, CERA‐treated cGN rats (gray bars). #*P* < 0.05 versus Sham control; *P < 0.05 versus cGN rats (*n* = 6–8). Values are expressed as mean ± SEM.

To assess whether CERA affect oxidative stress in the initial phase of the kidney disease, we measured urinary 8‐OHdG and L‐FABP levels over time. As shown in Figure 4, these oxidative stress markers were elevated at day 4. On the other hand, a single dose of CERA significantly alleviated them.

#### Changes in iron accumulation, plasma and urine iron, and iron‐related parameters in the kidney

**Figure 5:**
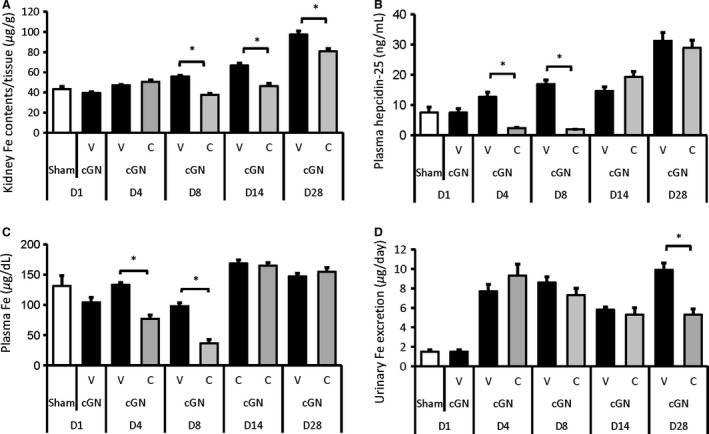
The effect of CERA on changes in (A) iron content per gram of kidney tissue, (B) plasma hepcidin‐25 levels, (C) plasma iron, and (D) urinary iron excretion from day 1 (D1) to day 28 (D28). Sham, sham‐operated control rats (white bars); V, vehicle‐treated cGN rats (black bars); C, CERA‐treated cGN rats (gray bars). #*P* < 0.05 versus Sham control; *P < 0.05 versus cGN rats (*n* = 6–8). Values are expressed as mean ± SEM.

In cGN rats, iron content in the kidney was significantly increased along with plasma hepcidin‐25 elevation (Fig. 5A & B). Iron content in the kidney gradually increased, and at the end of the study, it was elevated to about 3 times normal level (week 20: Sham, 96.5 ± 5.1 *μ*g/g; cGN + V, 328.9 ± 9.4 *μ*g/g; *P *<* *0.05). Urinary iron excretion was markedly elevated from day 4 and remained elevated until day 28 while plasma iron was not changed (Fig. 5C & D). On the other hand, CERA treatment significantly suppressed the increased kidney iron content and plasma iron accompanied by suppression of plasma hepcidin‐25. Of note, urinary iron excretion increased again in association with the increased tubular dilation in cGN rats. CERA treatment significantly suppressed the increased urinary iron excretion and tubular dilation.

#### Effect of CERA on changes in Ki67‐positive cells in glomeruli and tubules in the renal cortex

**Figure 6:**
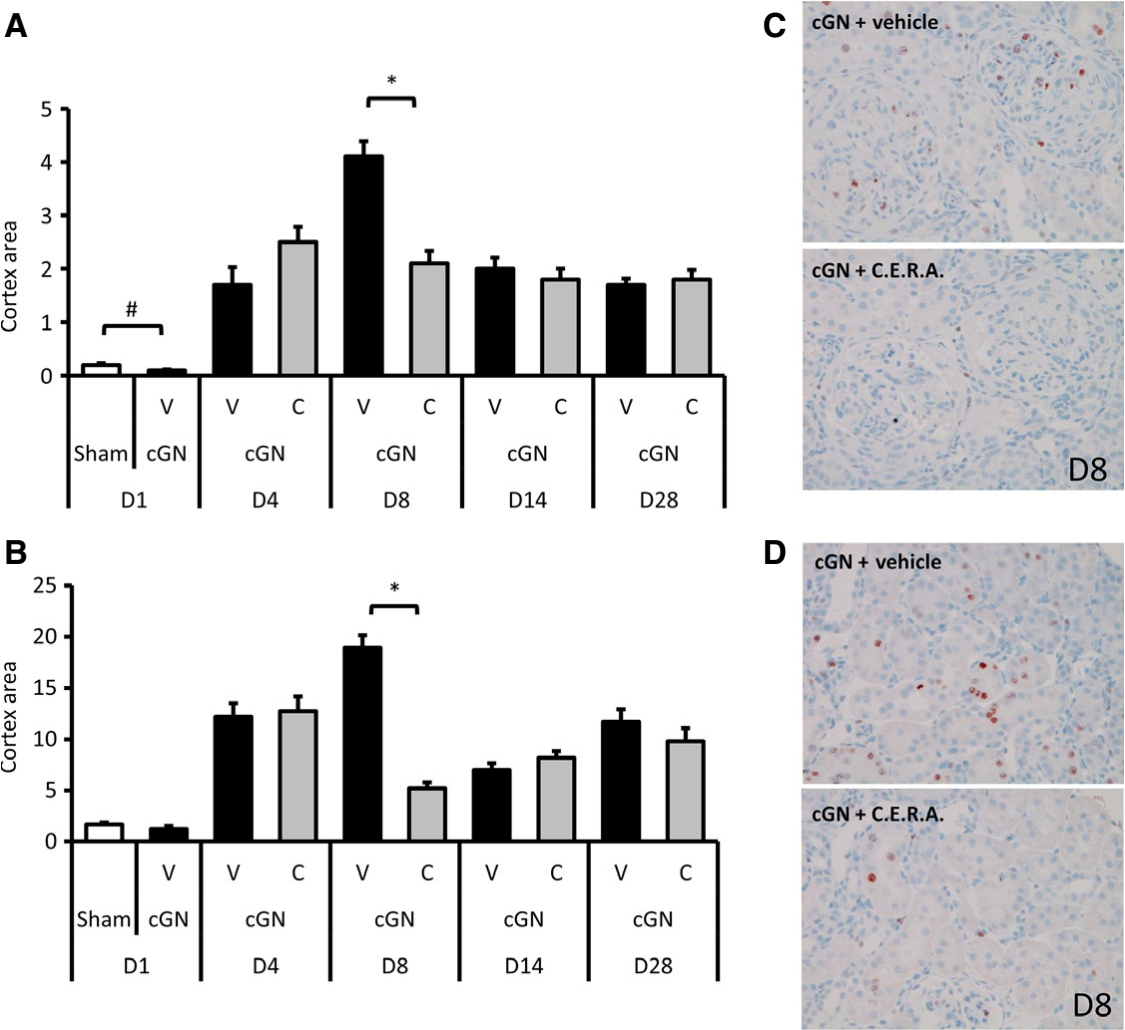
The effect of CERA on changes in Ki67‐positive cells in (A) glomeruli and (B) tubules from day 1 (D1) to day 28 (D28). Sham, sham‐operated control rats (white bars); V, vehicle‐treated cGN rats (black bars); C, CERA‐treated cGN rats (gray bars). Representative sections of Ki67 immunostaining of kidneys: (C) glomeruli and (D) tubular epithelium of renal cortex. Magnification ×280. #*P* < 0.05 versus Sham control; *P < 0.05 versus cGN rats (*n* = 6–8). Values are expressed as mean ± SEM.

To evaluate the effect of CERA on proliferation of kidney cells after the insult of kidney damage, we evaluated Ki67‐positive cells in glomeruli and tubular cells (Fig. 6). In cGN rats, the number of Ki67‐positive cells in both glomeruli and tubules of the renal cortex was markedly elevated and peaked at day 8, but these elevated counts did not continue to day 14. CERA treatment significantly inhibited the elevation. Of note, the number of Ki67‐positive cells in tubules (but not in glomeruli) began to increase again from day 14.

#### Correlation between kidney iron accumulation and parameters for tubular damage

**Figure 7:**
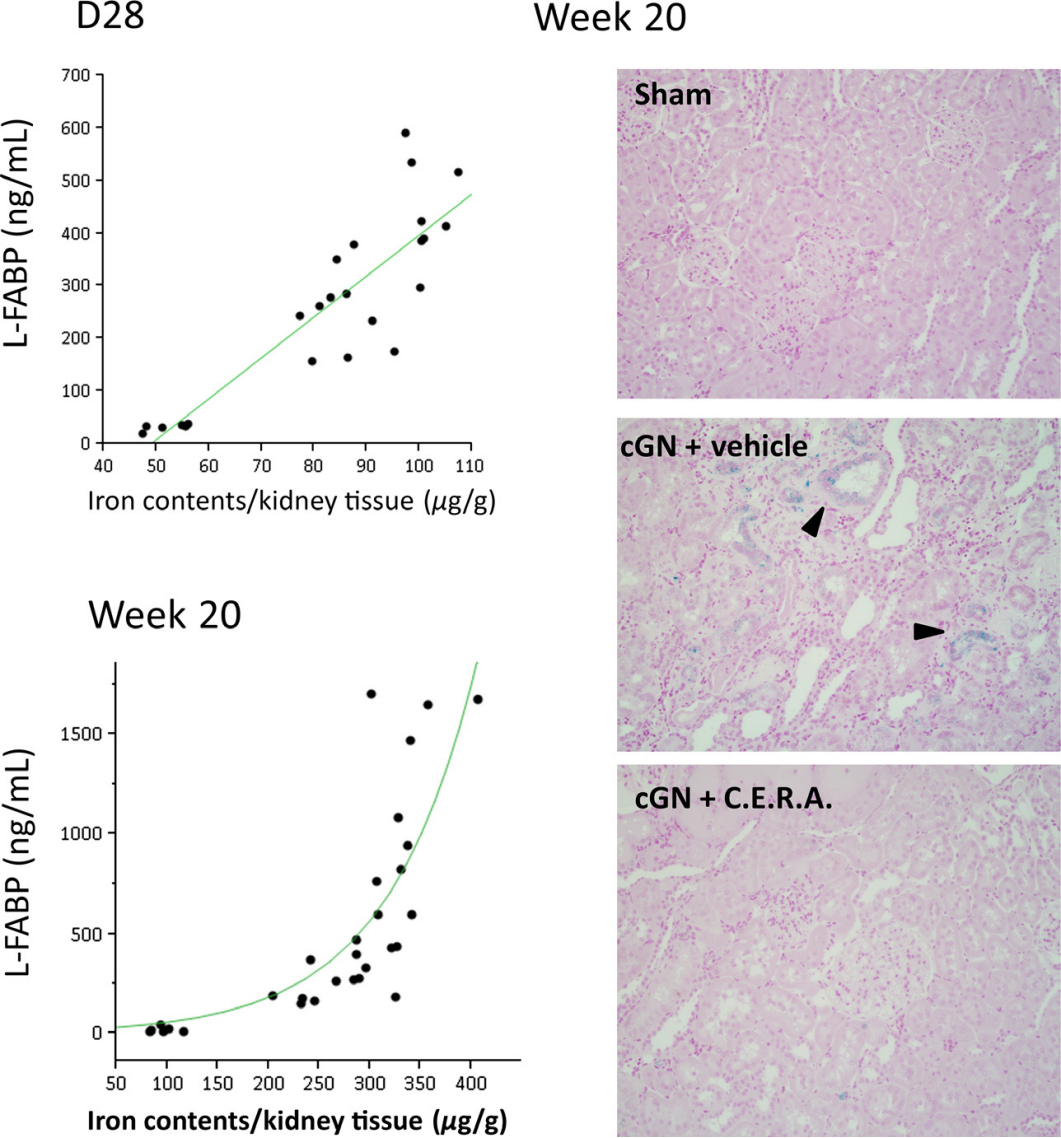
Correlation between iron content per gram of kidney tissue and urinary L‐FABP. Changes in iron content in kidney tissue and urinary L‐FABP at day 28 (D28) or week 20. Representative histological findings of iron staining (arrowheads) in Berlin blue staining section. Magnification ×280.

We assessed the correlation between kidney iron accumulation and L‐FABP at day 28 and week 20. Urinary L‐FABP levels were strongly correlated with iron deposition in the kidney at both day 28 and week 20 (Fig. 7). Iron deposition was mainly observed in tubular cells (Fig. 7).

## Discussion

In the present study, a single dose of CERA given on day 1, but not day 16, in a rat model of CKD was found to mitigate the initial glomerular injury, oxidative stress, and the deposition of iron in the kidney, thereby mitigating exacerbation of kidney damage and delaying progression of CKD.

There is a growing body of evidence derived from several models that the renal therapeutic benefits of EPO go beyond simply ameliorating anemia (Fliser et al. 2006; Bahlmann and Fliser 2009). A single injection of EPO has been shown to exert a renoprotective effect in some acute kidney disease models, but it had not been fully clarified whether that ability could extend to halting progression of chronic kidney disease. To address this issue, we selected a unique model with which we could evaluate the progression of CKD after applying treatment only in the acute phase of the disease. In fact, it has been reported that an initial treatment with anti‐PDGF antibody could halt progression of CKD in this model (Ostendorf et al. 2001; Tsuji et al. 2006).

We first evaluated the histopathological changes occurring after treatment with CERA in the initial phase of this model and noted that although an initial renoprotective effect by CERA was not clearly observed in the histopathological analysis (Fig. 3), one of the main tubular structural changes, tubular dilation, deteriorated again along with the exacerbation of uTP and UN. In addition, a single dose of CERA given on day 16 could not mitigate the deterioration of CCr. Therefore, to evaluate the mechanisms underlying the effect of CERA in retarding progression of CKD, we focused on the initial phase of kidney disease in our model.

We have already reported that CERA suppressed uTP excretion in acute glomerular disease model (Aizawa et al. 2014). Therefore, we first evaluated whether CERA could suppress uTP levels in acute phase of this model (day 2 and day 4). However, a single dose of CERA given on day 1 did not affect uTP excretion at any sampling points (data not shown). In the present study, we, therefore, evaluated the levels of urinary L‐FABP, a renoprotective protein localized predominantly in the proximal tubules, which has antioxidant properties (Wang et al. 2005). On day 4, urinary L‐FABP was significantly elevated and remains high until week 20 in cGN rats. CERA suppressed the elevated L‐FABP levels (Fig. 5). Therefore, we believed that main target of CERA for renoprotection might be tubules rather than glomeruli in our irreversible model.

In patients with kidney disease, renal tubules are exposed to high concentrations of iron owing to increased glomerular filtration of iron and iron‐containing proteins. This increased iron load is associated with accumulation of iron in the kidneys of patients with glomerulopathy (Shah et al. 2007; Moreno et al. 2012). It has been reported that in rats with proteinuria induced by glomerular disease, iron accumulation in the proximal tubules could possibly be a predictor of both functional and structural damage in the proximal tubules (Harris et al. 1994). In our present study, urinary iron excretion was significantly elevated after renal insult and subsequent iron content in the kidneys of cGN rats increased along with glomerulopathy. Excessive urinary iron or other proteins excretion induce oxidative stress as indicated the elevation of oxidative stress markers, 8‐OHdG and L‐FABP, and resulted in the elevation of iron contents in kidney. Furthermore, Ki67‐positive cells gradually increased in the tubules of the renal cortex coincide with the iron accumulation in kidney and then diminished although iron deposition increased. On the other hand, CERA suppressed these oxidative stress markers at day 4 and inhibited the subsequent growth of Ki67‐positive cells with the elevation of iron contents in kidney at day 8. However, we have not testified CERA treatment could mitigate iron‐mediated oxidative stress because CERA did not inhibit iron contents in kidney at day 4. Therefore, the further study whether CERA can suppress oxidative stress directly or through other factors would be needed. The impediment of tubular cell growth by CERA might be partly involved in the exacerbation of kidney dysfunction from day 14. In the reversible model of anti‐Thy1 nephritis (Canadillas et al. 2010), darbepoetin‐*α* could accelerate glomerular cell growth, but according to our findings using the irreversible model of anti‐Thy1 nephritis, promotion of glomerular cell growth was not observed. Thus, the renoprotective effect of CERA in our model may be associated with ameliorating damage in tubules rather than in the glomeruli. Taken together, these results suggested that both the alleviation of iron deposition in the kidney and increased cell growth resulted in promoting tubular regeneration, thereby protecting against CKD progression.

Several mechanisms for the renoprotective effect shown by single doses of ESAs in acute kidney disease models have been reviewed (Fliser et al. 2006). ESAs are able to protect against initial kidney damage caused by ischemic insult or induced by glomerulopathy by alleviating apoptotic activity or oxidative stress (Johnson et al. 2006; Canadillas et al. 2010). Katavetin et al. demonstrated that EPO induced heme oxygenase (HO)‐1 to attenuate oxidative stress, which might be associated with slowing CKD progression in Dahl‐salt sensitive rats (Katavetin et al. 2007). In our model, the expression of HO‐1 protein in the renal cortex was significantly elevated at day 4. However, CERA treatment did not influence HO‐1 protein (data not shown). Thus, delaying CKD progression by a single dose of CERA could be partly due to attenuation of oxidative stress, but did not involve the induction of HO‐1 in our study.

In the clinical setting, whereas results of smaller randomized clinical studies (Kuriyama et al. 1997; Gouva et al. 2004) have suggested that anemia correction with recombinant human EPO could slow progression of CKD, data from large trials have revealed no such beneficial effect (Drueke et al. 2006; Singh et al. 2006). Thus, the clinical benefit of ESAs on protecting against CKD progression still remains unresolved. Nevertheless, our study provides basic evidence that CERA can exert a beneficial effect. Future studies are expected to clarify the best protocol under which to use ESAs to exert renoprotection and also to develop specific biomarkers to indicate when such treatment is likely to be successful.

In conclusion, a single dose of CERA could mitigate exacerbation of kidney damage, thereby delaying CKD progression in a glomerulonephritis rat model. CERA treatment suppressed oxidative stress, which would be involved in help regeneration of tubular cells. In addition, the alleviation by CERA of the exacerbation of kidney damage could be attributable to mitigation of tubular damage as a result of lowered iron deposition in the tubules.

## Conflict of Interest

All authors are employees of Chugai Pharmaceutical Co., Ltd.
